# The VAAST Variant Prioritizer (VVP): ultrafast, easy to use whole genome variant prioritization tool

**DOI:** 10.1186/s12859-018-2056-y

**Published:** 2018-02-20

**Authors:** Steven Flygare, Edgar Javier Hernandez, Lon Phan, Barry Moore, Man Li, Anthony Fejes, Hao Hu, Karen Eilbeck, Chad Huff, Lynn Jorde, Martin G. Reese, Mark Yandell

**Affiliations:** 1 Department of Human Genetics, University of Utah, Salt Lake City, UT USA; 2 USTAR Center for Genetic Discovery, Salt Lake City, UT USA; 3 National Center for Biotechnology Information, Bethesda, MD USA; 4 Department of Epidemiology, M.D. Anderson Cancer Center, Houston, TX USA; 5 Fabric Genomics, Oakland, CA USA; 6 Department of Biomedical Informatics, University of Utah, Salt Lake City, UT USA; 7 Present address: IDbyDNA Inc., San Francisco, CA USA

**Keywords:** Variant prioritization, Genomics, Human genome, Variants of uncertain significance

## Abstract

**Background:**

Prioritization of sequence variants for diagnosis and discovery of Mendelian diseases is challenging, especially in large collections of whole genome sequences (WGS). Fast, scalable solutions are needed for discovery research, for clinical applications, and for curation of massive public variant repositories such as dbSNP and gnomAD. In response, we have developed VVP, the VAAST Variant Prioritizer. VVP is ultrafast, scales to even the largest variant repositories and genome collections, and its outputs are designed to simplify clinical interpretation of variants of uncertain significance.

**Results:**

We show that scoring the entire contents of dbSNP (> 155 million variants) requires only 95 min using a machine with 4 cpus and 16 GB of RAM, and that a 60X WGS can be processed in less than 5 min. We also demonstrate that VVP can score variants anywhere in the genome, regardless of type, effect, or location. It does so by integrating sequence conservation, the type of sequence change, allele frequencies, variant burden, and zygosity. Finally, we also show that VVP scores are consistently accurate, and easily interpreted, traits not shared by many commonly used tools such as SIFT and CADD.

**Conclusions:**

VVP provides rapid and scalable means to prioritize any sequence variant, anywhere in the genome, and its scores are designed to facilitate variant interpretation using ACMG and NHS guidelines. These traits make it well suited for operation on very large collections of WGS sequences.

**Electronic supplementary material:**

The online version of this article (10.1186/s12859-018-2056-y) contains supplementary material, which is available to authorized users.

## Background

Variant prioritization is the process of determining which variants identified in the course of genetic testing, exome, or whole-genome sequencing are likely to damage gene function (for review [1–3]). Variant prioritization is central to discovery efforts, and prioritization scores are increasingly used for disease diagnosis as well. Both the American College of Medical Genetics and National Health Service of the United Kingdom have published guidelines for employing prioritization scores during clinical review of variants of unknown significance, or VUS [4–6].

The advent of whole genome sequencing (WGS), along with ever-growing clinical applications, has produced a host of new bioinformatics challenges for variant prioritization. Ideally, a tool should compute upon any type of variant, scale to large discovery efforts, and integrate the diverse data types that inform the prioritization process. Its scores also need to be intelligible to clinical genetics professionals. Meeting all of these requirements with a single tool is no easy matter.

Another challenge is how best to incorporate population and gene-specific variation rates into prioritization scores. The density of variation is not constant within a gene; for example, intronic variation is more frequently observed than exonic [7–9]. Moreover, the amount of potentially damaging variation varies between genes, a phenomenon referred to as ‘burden’ [2, 10]. Zygosity is another source of information for prioritization; logically, a likely damaging variant is more likely to be pathogenic when homozygous.

Speed is also an issue. Rapid prioritization of the many millions of sequence variants found in large collections of WGS is a challenging problem. One approach is to cache previously seen variants [11]. This is effective when processing a single genome or small cohort. However, because most sequence variation is rare [7–9], large cohorts can contain millions of new variants that have not been seen before. Maintaining reasonable run times on WGS datasets, while effectively integrating the heterogeneous data types required for prioritization, is an informatics challenge.

VVP employs variant frequencies as an *observable* in its calculations by means of a likelihood-ratio test. As we show, this big-data approach allows it to directly leverage information in public variant repositories for variant prioritization. This means VVP can even use the contents of variant repositories to prioritize the repositories themselves. This has far reaching ramifications as regards scope of use. And, as we demonstrate, this simple approach is highly accurate. VVP integrates sequence conservation, the type of sequence change, and zygosity for still greater accuracy.

VVP is also designed to simplify and speed variant interpretation. VVP scores are designed for optimal utility for discovery and interpretation workflows that employ score-based filtering. Moreover, VVP scores also make it possible to compare the relative impact of different variants within and between genes. VVP scores facilitate these use-cases because they are consistently accurate across their entire range, a trait not shared by commonly used tools. As we show, these features of VVP scores greatly simplify and empower interpretation of Variants of Uncertain Significance (VUS) using ACMG and NHS guidelines [4–6].

Finally, VVP is very fast. A 60X WGS can be processed in about 4 min using 4 cpus and 16 GB of RAM, which is within the range of typical laptop computers. To demonstrate VVP’s utility we used it to prioritize the entirety of dbSNP [12], some 155 million variants, in 95 min using a computer with 4 cpus and 16 GB of RAM.

## Methods

### Raw scores

The VAAST [13] Variant Prioritizer (VVP) can assign a prioritization score to any type of sequence variant, located anywhere in the genome. To do so, VVP leverages the same Composite Likelihood Ratio Test (CLRT) used by VAAST [13] and its derivatives, VAAST 2.0 [14] and pVAAST [15]. Whereas those tools use the CLRT to score genes to perform burden-based association testing in case-control and family based analyses [2, 16], VVP reports scores for individual variants, and is designed for very large-scale variant prioritization activities. Run times are a major motivation for the VVP project, which is why VVP is written entirely in C, including the VCF parser. All of these factors combine to allow VVP to score every variant in a typical WGS in less than 5 min using a computer with just 4 cpus and 16 GB of RAM.

VVP places two scores on each variant: a raw score and a percentile score. Variant genotype is fundamental to the VVP scoring process, and VVP provides a score for a variant in both the heterozygous and homozygous state. As we show, doing so facilitates and speeds variant interpretation.

Raw scores (λ in Eq. 1) are calculated using the VAAST Likelihood Ratio Test (LRT) [13, 14].

The LRT calculation

1 $$ \lambda =\mathit{\ln}\left(\frac{Lnull}{Lalt}\ast \frac{h_i}{a_i}\right). $$

The numerator of the LRT is the null model (variant is non-damaging); the denominator is the alternative model (variant is damaging). The *ln* ratio between these models is the variant’s raw score. In eq. 1, the first component of the numerator (null model) is the likelihood of observing 1 (heterozygous) or 2 (homozygous) copies of the variant in a background distribution of N individuals sampled randomly from the population. The first component of the denominator (alt model) is the likelihood of observing 1 (heterozygous) or 2 (homozygous) copies of the variant under the assumption that the background data and the variant are derived from two distinct populations, each with its own frequency for the variant, e.g. the background population is ‘healthy’ (or more properly speaking, has been drawn randomly from the population) and the case population is comprised of one or more affected individuals. The key assumption here is that deleterious variants tend to be minor alleles, because they are under negative selection. For example, the theoretic population equilibrium frequency for a deleterious variant with a negative selection coefficient of 0.01 is 2.2 × 10^− 4^ [13, 15].

The LRT in expanded form

2 $$ \lambda =\mathit{\ln}\left[\frac{p^x{\left(1-p\right)}^{n-x}}{{p_u}^{x_u}{\left(1-{p}_u\right)}^{n_b-{x}_u}{p_a}^{x_a}{\left(1-{p}_a\right)}^{n_t-{x}_{\mathrm{a}}}}\ast \frac{h_i}{a_i}\right]. $$

Equation 2, shows the LRT in expanded form. Here *x* is the number of chromosomes in the proband(s) with that variant, *n* is the total number of chromosomes in the proband(s) and population combined, and *p* is frequency of the variant in the probands(s) and population combined. *x*_*u*_ is the total number of chromosomes bearing the variant allele in the population, *n*_*b*_ is the total number of chromosomes in the population, and *p*_*u*_ is the population allele frequency. *x*_*a*_ is the number of chromosomes bearing the variant in the proband(s), *n*_*t*_ is the number of chromosomes in the probands(s), and *p*_*a*_ is the variant frequency in the proband(s). *N choose x* terms from the binomial formulas are constants and have be removed from Eq. 2. *a*_*i*_ and *h*_*i*_ parameterize the variant effect as in Eq. 1.

Putting aside *a*_*i*_ and *h*_*i*_, for the moment, note that Eq. 2 employs variant frequencies directly as *observables*. This approach has interesting ramifications as regards cross validation. Consider that the maximal impact of including or excluding a proband from the population data used in its calculations is proportional to (n - c)/(x - c), where n is the observed count of the variant in the population, x is the number of chromosomes in the population dataset, and c is the count for the proband genome, i.e. 1 or 2, depending on zygosity. Now consider that gnomAD currently contains 15,496 whole genomes, therefore x = 30,972. Because (n - c)/(x – c) ≈ n/x, lambda is little changed regardless of whether or not a given proband is included or excluded from the population dataset. Changes to lambda are further buffered by the percentile scoring method described below. Consistent with these observations, removing all NA12878 variants from gnomAD, increases the VVP pathogenic call rate on NA12878 for coding variants from 4% to 4.2%. The call rate for non-coding variants is unchanged. These facts illustrate the utility of treating variant frequency as an observable, and show how the scale of today’s repositories accommodates VVP’s big-data approach. At these scales, VVP can even prioritize the contents of variant repositories themselves, which has far reaching ramifications as regards scope of use. For example, in collaboration with the National Center for Biotechnology Information (NCBI), we have used VVP to score the entire contents of dbSNP, some 155 million variants. Using a machine with just 4 cpus and 16 GB of RAM this took 95 min.

For the analyses presented here, population variant frequencies were compiled from the WGS portion of gnomAD (gnomad.broadinstitute.org/). These data are also distributed with VVP in a highly-compressed format. Users may also create their own frequency files using private and/or other public genome datasets. Details are provided in the VVP GitHub repository.

VVP also models variant ‘consequence’ or ‘effect’, as this has been shown to improve performance [3, 11, 13, 14, 17]. VVP does so using annotation information stored in the info field of VCF formatted variant files [18]. VVP uses the following annotation information: transcript id, Sequence Ontology terms, and amino acid change [19, 20]. Annotation tools like VEP and VAT, the VAAST Variant Annotation Tool, can provide the annotations required by VVP [13, 16, 21]. Although annotations are not strictly required, their use is recommended. For the analyses described here, variant effects were determined using Ensembl gene models and VEP. Because VVP is entirely vcf-based, workflows are very simple, e.g. vcf- > VEP- > VVP.

Variant impact is modeled using two parameters, *h*_*i*_ and *a*_*i*_ (see Eqs. 1 and 2). *h*_*i*_ is equal to the frequency of a given *type* (*i*) of amino acid change in the population. The parameter *a*_*i*_ in the alternative model (denominator) is the observed frequency of that *type* of change among known disease-causing alleles We previously estimated *a*_*i*_ by setting it equal to the proportion of each *type*_*i*_ of amino acid change among all known disease-causing mutations in OMIM and HGMD [13, 14]. The same approach was used for modulo 3 and non-modulo 3 indels. Details of the approach can be found in the methods sections of those publications. The key concept here is that VVP, like VAAST, models impacts by *type*, e.g., how often are R- > V missense variants observed (in any gene, at any location, in any genome) within gnomAD genomes (*h*_*i*_) compared to how frequently they are observed a dataset of known disease-causing variants (in any gene, at any location), *a*_*i*_. See our previous publication [14] for more on these points. As is the case for variant frequencies, these values are little affected by the presence or absence of a particular variant instance having been observed in OMIM, ClinVar, or gnomAD. Consider that once again, the effect is proportional to (n - c)/(x - c), only here, for *a*_*i*_, n is the observed count of R- > V missense inducing variants in OMIM and HGMD, and x is the total number of different variants in OMIM and HGMD. For *h*_*i*_, n is the total number of different R- > V missense inducing variants in gnomAD, and x is the total number of different sequence variants in gnomAD. For our ClinVar benchmarks c = 1. Because n and x are even larger for impact calculations than they are for VVP’s variant frequency calculations, including or excluding a particular variant in the calculations has even less effect on impact scores than it does for variant frequencies. These changes to lambda are further buffered by the percentile scoring method described below. Once again, this shows how VVP is designed to leverage big-data, and why its scope of use is potentially so broad.

The parameters *a*_*i*_ and *h*_*i*_ also incorporate information about phylogenetic conservation. This is taken into account for both coding and non-coding variants using PhastCon scores [22], another direct observable. Further details about how *h*_*i*_ and *a*_*i*_ incorporate this component into the LRT calculation can be found in [13, 14].

Alternatively, a Blosum matrix [23], rather than OMIM and HGMD can be used to derive *h*_*i*_ and *a*_*i*_, with Blosum matrix values used to determine missense impact. The process and resulting performance is described in [13]. Impact (*a*_*i*_ and *h*_*i*_) can also be removed completely from VVP’s calculations, meaning that variants can be prioritized using only variant frequencies. VVP users can invoke these different impact scoring methods, or turn them off entirely using command line options.

In order to assess what role, if any, the source of parameters *h*_*i*_ and *a*_*i*_ played in VVP’s performance on the ClinVar benchmarks reported below, we benchmarked VVP using (1) OMIM/HGMD with PhastCon scores; (2) using Blosum derived values for amino-acid substitutions only; and (3) with impact scoring turned off entirely (Additional file 1: Figure S1). As can be seen, VVP still matches or out performs commonly used tools such as CADD [11] and SIFT [17], regardless of which process is used to derive *a*_*i*_ and *h*_*i*_, even when impact scoring is turned off entirely. These results demonstrate how variant frequency at big-data scales can provide simple and powerful means for variant prioritization, and that the likelihood ratio test (Eqs. 1 & 2) effectively converts an observed variant frequency into a meaningful variant prioritization score. The calculation is simple, and as we show below highly accurate and very fast.

### Percentile scores

**Fig. 1:**
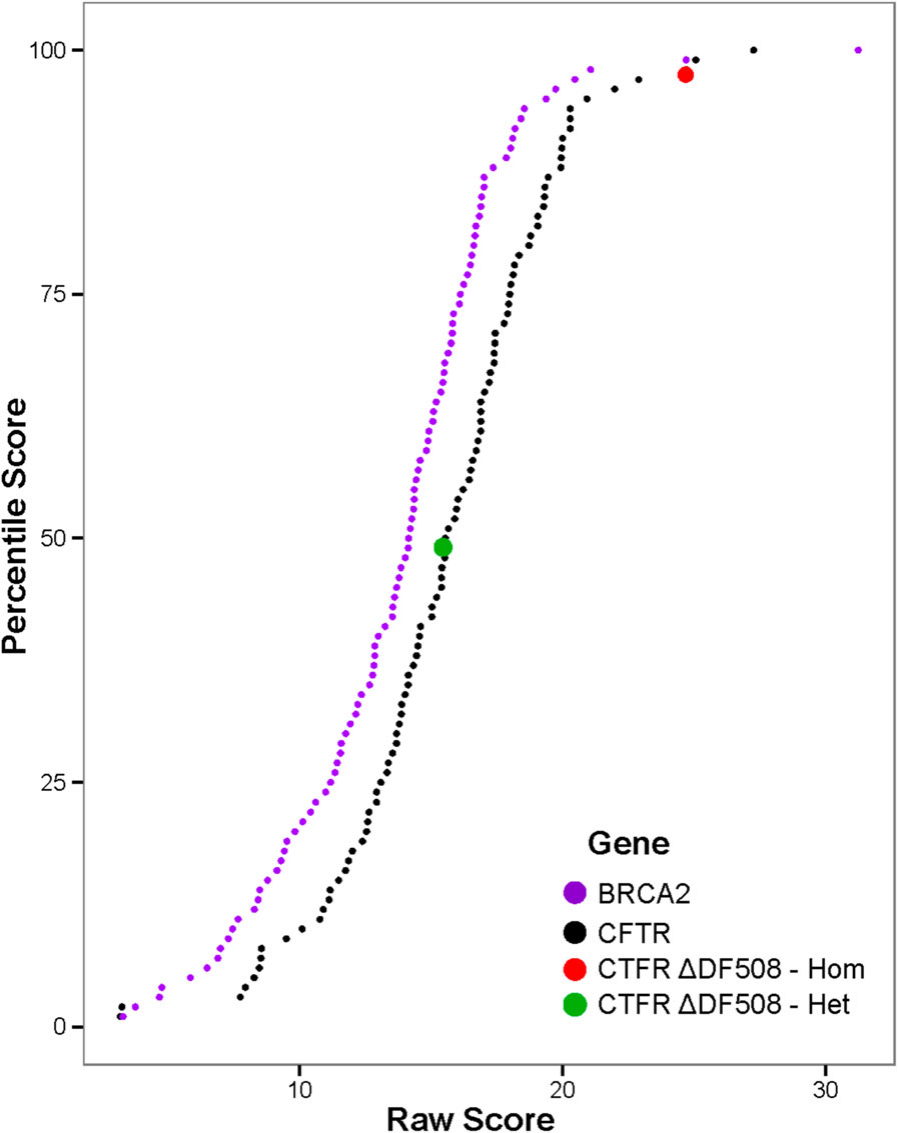
CRD curves normalize raw scores across genes. VVP raw score CRD curves for BRCA2 (purple), and CFTR (black), respectively. Note that a given CFTR raw score achieves a lower percentile score than does the same raw score for BRCA2. Red and green dots correspond to the canonical pathogenic CFTR variant ΔF508 scored as a homozygote and heterozygote, respectively

To facilitate variant interpretation, VVP raw scores are re-normalized on a gene-by-gene basis to generate VVP percentile scores. These percentile scores range from 0 to 100 and take into account differences in gene-specific variation rates (burden [2]) within the population. Percentile scores are generated as follows. First, VVP is used to score the entire contents of a variant repository to be used as a background. For the analyses presented here, we used the gnomAD whole genome vcf data. VVP requires only hours to build a reusable database based on gnomAD using 20 cpus and 20 GB of RAM. Next, VVP raw scores (*λ*) for every variant observed in the population (gnomAD) are then grouped according to the gene in which they reside. These gene-specific sets of variants are then further categorized in the VVP database into *effect groups* [1] coding (missense, stop-gained, splice-site variants, etc.) and [2] non-coding (intronic, UTR and synonymous variants). The remaining intergenic variants comprise the third category. Next, the coding variants in each gene are used to construct a cumulative rank distribution (CRD) for each gene, with raw scores on the x-axis and their percentile ranks on the y-axis. The same procedure is also used to construct a non-coding CRD for each gene. Finally, all remaining intergenic variants are grouped into a single intergenic CRD. The VVP raw scores are then renormalized to percentile ranks using these lookups. This renormalization greatly eases interpretation, as percentile scores provide a means to assess the relative severity of a variant compared to every other variant observed in the background population for that gene. Percentile scores also make it possible to compare the relative predicted severity of two variants in two different genes despite differences in gene-specific variation rates. Figure 1 illustrates this process for two genes, CFTR and BRCA2.

## Results & discussion

### Run times

Table 1 compares VVP runtimes to those of CADD v. 1.3 [11]. Like CADD, VVP is designed for WGS sequences and can score SNVs, INDELS and both coding and non-coding variants. We benchmarked VVP runtimes using a cohort of 100, 1000, and 10,000 variants by randomly selecting them from the 1000 Genomes Project phase 3 VCF file (All chromosomes, 2504 individuals). These files were then processed by VVP and CADD on the same machine and the runtimes were recorded. All relevant CADD cache files were downloaded to maximize performance. We ran CADD according to the instructions in the download bundle from the CADD website and recorded its processing time. As can be seen, VVP is much faster than CADD. One reason for this may be that CADD, like VVP, uses VEP annotations in its scoring. For VVP, VEP is run prior to scoring, so that this pre-compute may be parallelized if desired. Thus, we do not include the VEP run time in our recorded run times. CADD provides no option to run VEP prior to processing the vcf file. Even after downloading all relevant cache files, CADD continues to run VEP (version 76) during its scoring process, which we suspect is a major contributor to its long run times. Another issue has to do with the speed of scoring. To mitigate this problem, CADD provides users with a large precomputed file of every possible SNV, and common INDELS from ExAC. The problem with this approach is that every time a new INDEL is encountered in their own data, users must run CADD on it. Since most variation is rare, especially for indels, this creates a compute bottleneck, with runtimes running to many hours for a single WGS.

**Table 1:** Runtimes. Seconds required by VVP and CADD to process 100, 1000, and 10,000 variants

Number of variants	VVP	CADD
1000	0.1	130.9
10,000	0.9	1388.5
100,000	8.2	12,716.3

### Accuracy

We used all pathogenic and benign variants from ClinVar [24] version 20,170,228 with one or more gold stars assigned for ‘Review Status’ to assess the accuracy of VVP and to compare it to SIFT [17] and CADD [11]. We also excluded from our analyses variants whose ClinVar CLNALLE value = − 1, indicating that the submitted allele is discordant with the current genome assembly and its annotations. There are 18,117 benign alleles and 14,195 pathogenic alleles in the resulting dataset. For the analyses presented herein, we used CADD v.1.3. For SIFT we used the values provided by CADD in its outputs. We compared those to VEP v.89 (which also provides SIFT scores), and to those provided by Provean [25]. The SIFT scores provided by CADD v1.3 resulted in equal or superior performance in our ROC analyses.

The widely used SIFT provides a basic reference point, as it has been benchmarked on many different datasets and compared to many different tools; likewise, the CADD primary publication [11] also presents numerous benchmarks. Thus, comparing VVP to these two tools provides means to relate its performance many other tools using a large body of previous work. Finally, use of Phenotype data for variant interpretation is becoming increasingly wide spread [26, 27], (see [2] for more on these points). Phevor [28], for example can use VVP percentile scores directly in its calculations and combine then with phenotype data [29].

**Fig. 2:**
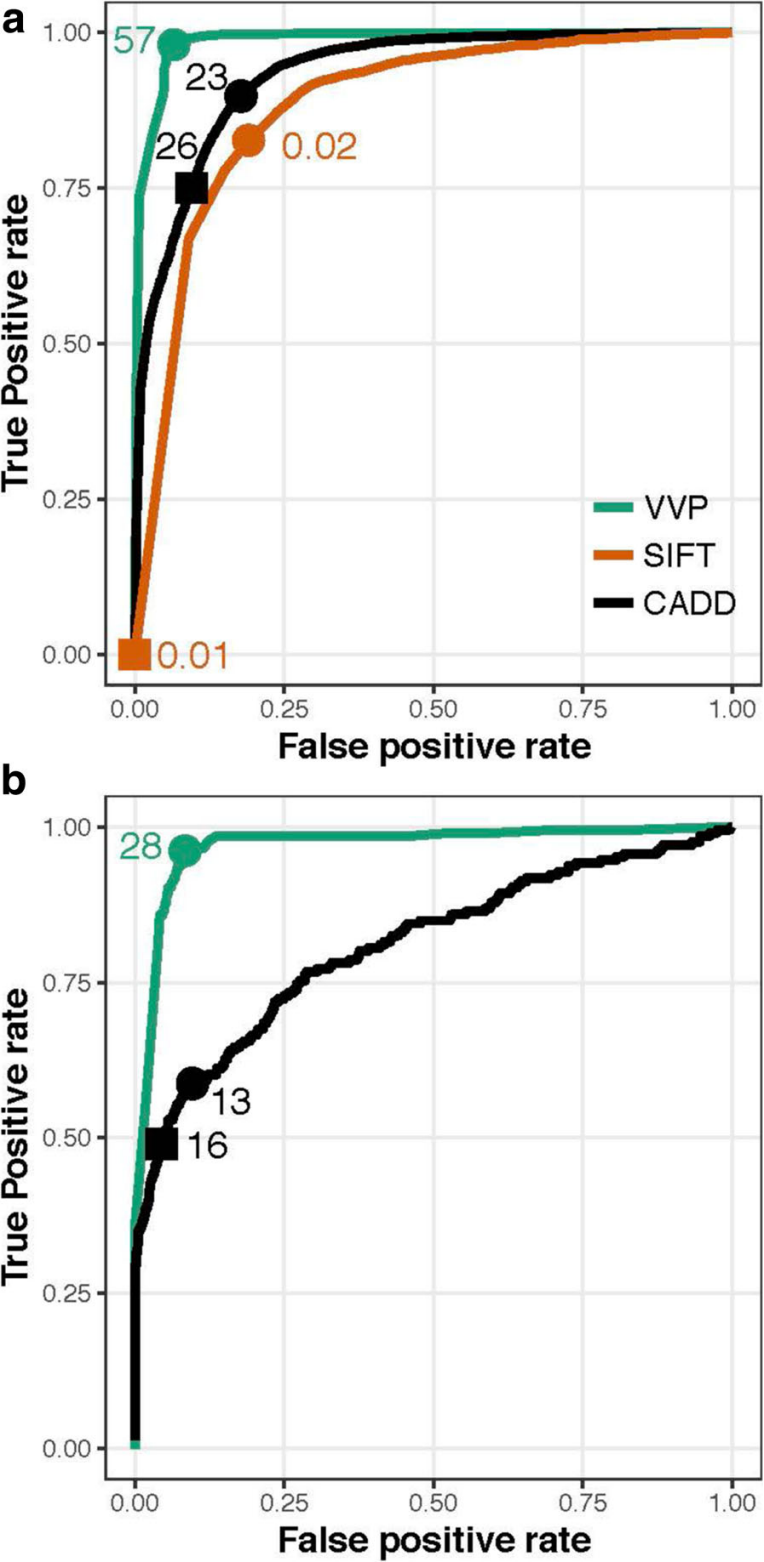
ROC analyses for ClinVar. **a** Coding Variants. **b** Non-coding variants. The points on the curves labeled with circles correspond to score thresholds resulting in each tool’s maximum accuracy. That score is shown beside the circle. Points denoted with squares correspond to the score threshold for SIFT and CADD required to reproduce VVP’s call rate for damaging variants on the NA12878 WGS. See Discussion and Table-3 for details. VVP was run using its default dominant model, whereby every variant is scored as a heterozygote. No data are shown for SIFT in panel B, as it does not score non-coding variants

Figure 2 shows the resulting ROC curves for all three tools for coding and non-coding variants. ClinVar variants not scored by SIFT were excluded from its ROC calculation. No curve is shown for SIFT in Fig. 2b as it does not operate on non-coding variants. For coding variants VVP’s AUC exceeds CADD’s (0.9869 vs. 0.9344). Both tools significantly outperform SIFT (0.8457). Also, labeled in Fig. 2a are points corresponding to each tool’s optimal threshold for distinguishing pathogenic from benign coding variants. For VVP, CADD, and SIFT these scores are 57 and 23, and 0.02 respectively. For VVP using its optimal score of 57 for coding variants, the true-positive rate is 0.9805 and the false positive rate is 0.0652. Parsing CADD at its optimal value [23] results in a TP rate of 0.8981, and a FP rate of 0.1776. Whereas, SIFT’s true positive rate is 0.8271, and its false-positive rate is 0.1905. Figure 2b shows performance for non-coding ClinVar Variants. Consistent with previous observations [30], CADD’s AUC for non-coding ClinVar variants is 0.8089, whereas VVP’s is 0.9695, demonstrating that VVP provides superior means for prioritizing non-coding variants.

### Youden’s J statistic

**Fig. 3:**
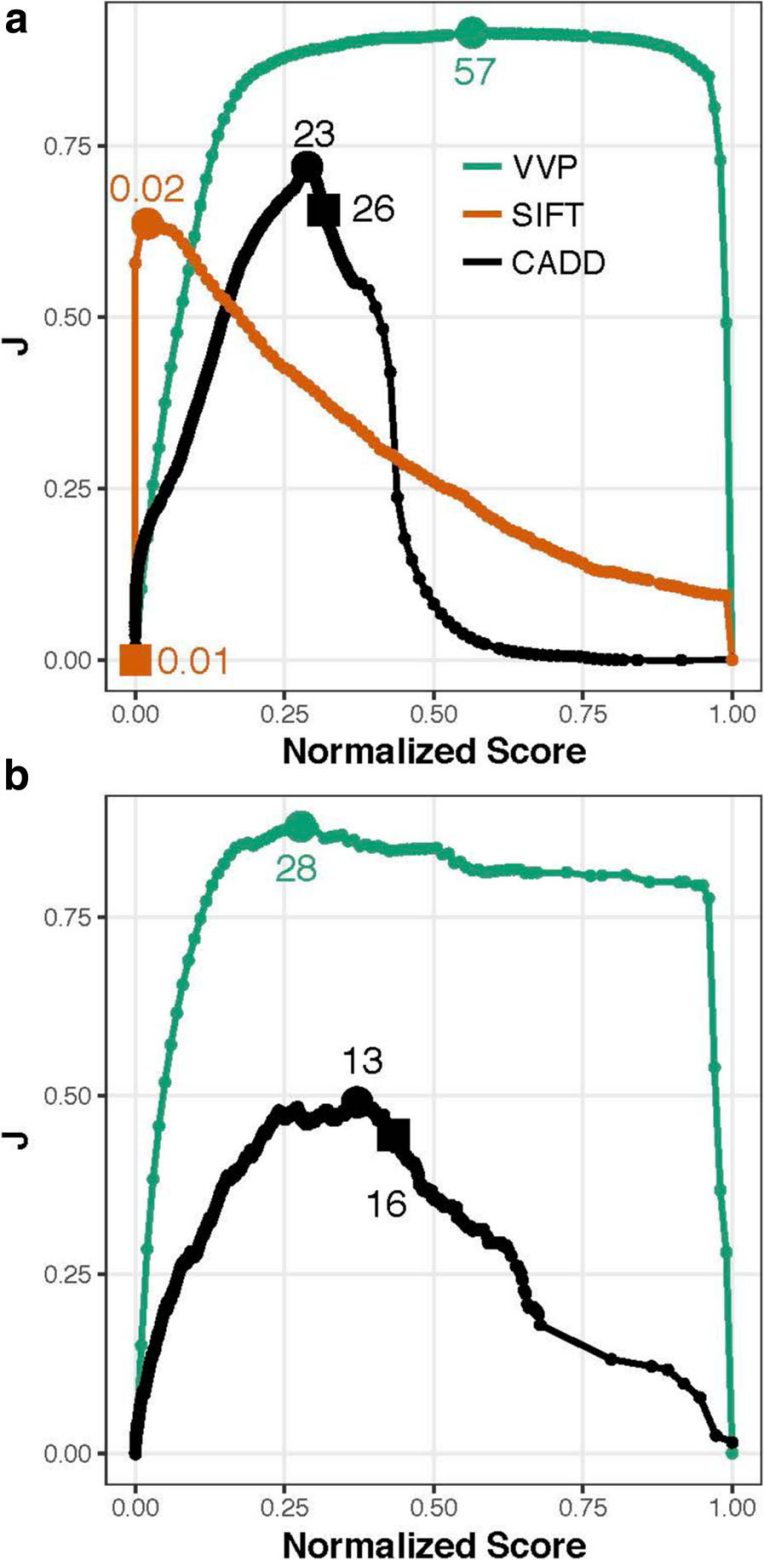
J curves for ClinVar. **a** Coding variants. **b** Non-coding variants. The units on the x-axis are percentile ranks for each tool’s score, i.e. score/max for each tool. Youden’s statistic (J) is plotted for each normalized score on the y-axis. As in Fig. 2, the points labeled with circles on the curves correspond to score thresholds resulting in each tool’s maximal accuracy. Squares denote score threshold to obtain VVP’s call rate on the NA12878. See Table-3 and Discussion for additional details. All tools were run using their recommended command lines. VVP J curves were compiled using percentile scores. No data are shown in **b**. for SIFT, as it does not score non-coding variants

Figure 3 shows the result of plotting Youden’s J statistic [31] for each tool using the same data and scores used in Fig. 2. J = sensitivity + specificity – 1. J values are also easily converted to accuracy, i.e. AC = (J + 1)/2, which provides familiar means to interpret the results in Fig. 3.

Youden’s statistic (J) is often used in conjunction with ROC curves because it provides means for summarizing the performance of a dichotomous diagnostic test, a topic not addressed by ROC analysis. While ROC analysis provides good means of summarizing overall performance of a tool, it says nothing about application accuracy, i.e. what happens when a given score is used as a threshold to distinguish positive from negative outcomes, e.g. pathogenic from benign variants. Clearly, employing a tool for variant interpretation requires one to make a decision based upon a score.

Importantly, Youden’s J statistic also provides means to assess the utility of filtering on a given score. A J value of 1 indicates that there are no false positives or false negatives, when choosing that threshold score, i.e. the test is perfect. A J of 0 indicates a test with no diagnostic power whatsoever, i.e. random guess. The ideal tool is one whose diagnostic value is perfect (J = 1) across the widest range of possible values.

The units on the x-axis in Fig. 3 are percentile ranks for each tool’s score, i.e. score/max for each tool. J is plotted for each normalized score on the y-axis. Plotting the scores in this way makes it possible to assess diagnostic value of each tool’s scores across their range, and compare tools to one another. Ideally J would be near one, and constant throughout the entire range of scores. As can be seen, for both coding and non-coding variants, VVP’s J curve is a close approximation of that ideal, except (as expected) at the limits, where sensitivity (x = 0) or specificity (x = 1) is zero. For coding variants, a VVP score of 20 has almost the same J value as one of 57. In contrast SIFT and CADD show very different behaviors.

Variant scores are routinely filtered to reduce the number of candidates in genome-based diagnostic activities [2]. To be effective, this activity relies upon assumption that a tool’s accuracy is constant across its range of scores, but as Fig. 3 makes clear, this is not necessarily the case. As can be seen, in contrast to SIFT and CADD, VVP’s accuracy is relatively constant across a wide range of scores. Moreover, there is no score on the SIFT and CADD curves that reaches the VVP optimum. Collectively, these two attributes mean that VVP scores have greater utility for discovery workflows that employ score-based filtering. Additional file 2: Figure S2, provides another view of these analyses that may be more intuitive to some readers. Recall that ClinVar variants are classified using a binary classification scheme: pathogenic or benign. In Additional file 2: Figure S2, scores are displayed as violin plots. Note that the pathogenic and benign distributions for SIFT and CADD overlap one another to a greater degree than do VVP’s. J-curves also have important ramifications for clinical variant interpretation, and the results shown in Fig. 3 demonstrate that VVP scores are also well suited for use in variant interpretation workflows such as those promulgated by the American College of Medical Genetics and National Health Service of the United Kingdom.

### Clinical utility

Table 2 shows clinical utility of each tool for the 10 genes in ClinVar with the most annotated pathogenic variants. Table 2 also gives the values for all ClinVar variants. We define clinical utility as accuracy multiplied by the fraction of variants scored. Thus, a tool that places a score on every variant, benign, pathogenic, coding and non-coding will have a clinical utility equal to its accuracy, i.e. (Sn + Sp)/2 at a given score threshold. Whereas, a perfectly accurate tool, that can only score half of the ClinVar variants, will have a global clinical utility of 0.5. SIFT, for example has a very low clinical utility for assessing BRCA2 alleles. This is because the majority of those variants are frameshifts and non-sense coding changes. SIFT does not score either class of variant, hence its utility for prioritizing BRCA2 variants is very low. Calculating accuracies in this way makes it possible to quantify the clinical utility a tool for scoring a specific gene, and for ClinVar as a whole. The data in Table 2 thus complement the ROC and J curves in Figs. 2 and 3, because for those figures we restricted our caculations to the variants scored by all three tools.

**Table 2:** Clinical Utility. Top panel. Gene-specific clinical utilities for the top ten ClinVar genes ranked by number of submitted variants. Bottom panel. Coding, non-coding and combined clinical utility for all ClinVar variants. Pathogenic thresholds for each tool were determined as in Fig. 3

Gene	VVP	CADD	SIFT
BRCA2	0.971	0.893	0.004
BRCA1	0.971	0.876	0.003
SCN1A	0.966	0.914	0.277
MLH1	0.943	0.950	0.057
MSH2	0.984	0.973	0.050
LDLR	0.989	0.890	0.033
DMD	0.959	0.932	0.030
ATM	0.957	0.953	0.021
FBN1	0.974	0.935	0.233
CFTR	0.945	0.930	0.073
Utility (All ClinVar Variants)			
Coding	0.970	0.900	0.792
Non-coding	0.917	0.715	0.000
Both	0.947	0.818	0.134

To identify the 10 genes highlighted in Table 2, we first excluded all ClinVar genes with fewer than 10 benign and/or pathogenic variants, and then ranked the remaining genes according to their number of ClinVar pathogenic variants. We also included CFTR, even though it has only 9 benign variants because of its clinical interest, and because it a focus of some of our discussions below (e.g. Fig. 5). The bottom panel of Table 2 also provides ClinVar-wide utility values for all variants, irrespective of gene**.** Because VVP and CADD score every variant, these values correspond to the peaks labeled in Figs. 2 and 3; this, however, is not the case for SIFT, and its values are correspondingly lower throughout. These results document gene-specific differences in clinical utility, with VVP outperforming the two other tools for clinically important genes such as CFTR, BRCA1 and BRCA2.

### WGS applications

Next, we benchmarked all three tools on the reference genome NA12878 WGS [32]. Our goal being to examine each tool’s behavior on an actual WGS. Since VVP is designed for such high-throughput operations, understanding this behavior is important. A tool, for example, might perform well on ClinVar, but have an unacceptable false positive rate when run on an actual exome or genome. For such applications, VVP’s superior J-curve is of paramount importance, because score-based filtering can be used to shorten the list of possible disease-causing variants, with little loss in accuracy. This is less true for CADD and SIFT (Fig. 3).

Even though ground truth is not known for this genome, collectively the results presented in Table 3 give some indication of the false-negative and false-positive rates of VVP compared to related tools when run the WGS of a presumably healthy individual.

**Table 3:** Call rates on reference genome NA12878, a healthy individual. Although the number of damaging coding and non-coding variants in a healthy individual’s genome is still unknown, presumably damaging variants comprise a low percentage of the total. Relative percentages are shown in the top panel, absolute numbers are shown in the bottom. Rare variants denotes variants with gnomAD population frequencies < 1/1000

	**All Variants (%)**			**Rare Variants (%)**		
**CODING**	**VVP**	**CADD**	**SIFT**	**VVP**	**CADD**	**SIFT**
**Pathogenic**	**4.0**	**11.1**	**13.2**	**23.5**	**31.7**	**24.5**
**Benign**	**96.0**	**88.9**	**58.1**	**76.5**	**68.6**	**56.9**
**Not Scored**	**0.0**	**0.0**	**28.7**	**0**	**0.0**	**18.6**
**NON-CODING**	**VVP**	**CADD**	**SIFT**	**VVP**	**CADD**	**SIFT**
**Pathogenic**	**1.7**	**3.5**	**0**	**43.23**	**4.33**	**0**
**Benign**	**98.3**	**96.5**	**0**	**56.77**	**95.67**	**0**
**Not Scored**	**0**	**0**	**100**	**0**	**0**	**100**
	All Variants (variants)			Rare Variants (variants)		
CODING	VVP	CADD	SIFT	VVP	CADD	SIFT
Pathogenic	577	1577	1883	48	64	50
Benign	13,710	12,710	8304	156	140	116
Not Scored	0.0	0.0	4100	0	0.0	38
NON-CODING	VVP	CADD	SIFT	VVP	CADD	SIFT
Pathogenic	31,079	64,571	0	3769	378	0
Benign	1,825,253	1,791,761	0	4949	8340	0
Not Scored	0	0	1,856,322	0	0	8718

For these analyses, NA12878 variants were derived from 1000 Genomes Project phase 3 calls. The data in Table 3 model an actual genome-wide application of each tool, a very different use-case from low throughput variant-by-variant prioritization common in diagnostic applications such as diagnosis using ACMG guidelines [4]. Even though ground truth is not known for this genome, collectively the results presented in Table 3 give some indication of the false-negative and false-positive rates of VVP compared to related tools when run the WGS of a presumably healthy individual. In total, there are 14,287 coding and 1,856,332 non-coding variants in the NA12878 WGS. It should be kept in mind that some percentage of its variant calls are errors. At these scales, the ability to accurately filter variants using scores to reduce the number of candidates is vital to many discovery and diagnostic workflows [2]. Once again, the J curves shown in Fig. 3 are of interest, as they provide means to access the accuracy of filter-based workflows.

To produce Table 3, VVP, SIFT and CADD were run using the same command lines and procedures used to create Figs. 2 and 3, and variants were classified as damaging or non-damaging using their optimal thresholds (see Figs. 2 and 3), Results are summarized for all variants and for rare ones (AF < 1/1000). Also recorded in Table 3 is the proportion variants not scored by a given algorithm. The bottom portion of Table 3 shows call rates non-coding variants. Variants from non-coding repetitive regions however been excluded using a RepeatMasker bed file from the UCSC genome Browser [http://genome.ucsc.edu/index.html].

Although the typical number of damaging coding and non-coding variants in a healthy individual’s genome such as NA12878 is still unknown, presumably damaging variants comprise a low percentage of the total. Consistent with this assumption, VVP identifies 4.0% of NA12878 coding variants damaging, whereas SIFT scores 8.5%, and CADD 11.1%. Consistent with previous reports [3], SIFT is unable to score some coding variants. Interestingly, this value changes with allele frequency (16.6% vs. 24.5%). This behavior is a consequence of the greater proportions of frameshifting and stop-codon inducing variants at lower allele frequencies (see discussion of Additional file 3: Figure S3, below). VVP and CADD also report higher percentages of rare variants as pathogenic due the same phenomenon.

If a tool has a well-behaved J-curve (Fig. 3), for WGS datasets, filtering on the tool’s scores will reduce the number of candidate variants without sacrificing accuracy. However, if the tool has a poorly behaved J-curve, score threshold-based filtering will be ineffective. To illustrate this point, we asked what score for each tool would result in the same NA12878 call rate as VVP’s for coding variants, e.g. 4.0%. That value for CADD is 26, and for SIFT is 0.01. These points are also labeled with squares on the curves shown in Figs. 2 and 3. Consider that in order to obtain VVP’s 4.0% pathogenic call rate on NA12878, SIFT would have a true positive rate of essentially zero for ClinVar data. In other words, the only way to obtain a 4.0% call rate on a WGS would be to invoke such a high score threshold for SIFT that its ClinVar TP rate would be zero. CADD exhibits similar behavior, although it is much less severe. Achieving a 4.0% call rate on NA1278 with CADD would require a score threshold of 26; that same score would result in a 0.74 TP rate on ClinVar (Fig. 2a), and its diagnostic accuracy, (Fig. 2b), would be 0.63. In contrast, VVP’s ClinVar TP rate would be 0.98, and its diagnostic accuracy would be 0.91. The same trends hold true for non-coding variants too. For example, increasing VVP’s non-coding threshold score for damaging non-coding variants from 28 to 75 would decrease the number of predicted rare pathogenic non-coding variants in NA12878 from 3769 to 152, and the percentage would drop from 43.23% to 1.74%. Again, the flat J-curve for non-coding variants (Fig. 3b) indicates that this would have minimal impact on overall accuracy.

These facts illustrate the demands placed on prioritization tools by WGS big-data, and the complexities and hidden assumptions introduced by score-based filtering approaches. We argue that the constancy of VVP’s performance characteristics for both diagnostic and big-data WGS applications is a major strength.

Additional file 3 Figure S3 shows that the results shown in Figs. 2, 3 and Table 3 reflect how (if at all) variant frequencies are handled in each tools’ prioritization calculations. Each panel in Additional file 3: Figure S3 plots mean score of a tool vs. binned allele frequency. All three tools (SIFT, CADD, and VVP) have negative slopes. As SIFT does not consider variant frequencies, its curve illustrates how phylogenetic sequence conservation varies inversely with variant frequency, and presumably the intensity of purifying selection (SIFT’s central assumption). Note that CADD’s curve is similar to SIFT’s, but has a more negative slope, improving performance. In contrast, VVP’s curve is highly non-linear, and common variants very rarely achieve pathogenic scores. Thus, these curves illustrate why for SIFT and CADD, so many variants with population frequencies > 5% are judged damaging, resulting in the high call rates for common variants seen for WGS sequences (Table 3). Additional file 4: Figure S4 and Additional file 5: Figure S5 break down every CADD call in ClinVar and NA12878 according to CADD consequence category and compare CADD’s scores to VVPs. These data demonstrate that stop gains and frameshifts are assigned high CADD scores, even when they are frequent in the population, a source of false positives when running CADD on a WGS dataset that VVP’s LRT approach mitigates. Collectively, Additional file 3: Figure S3 and Additional file 4: Figure S4 and Additional file 5: Figure S5 further illustrate the importance of variant frequency for prioritization.

### VVP scores for dbSNP

**Fig. 4:**
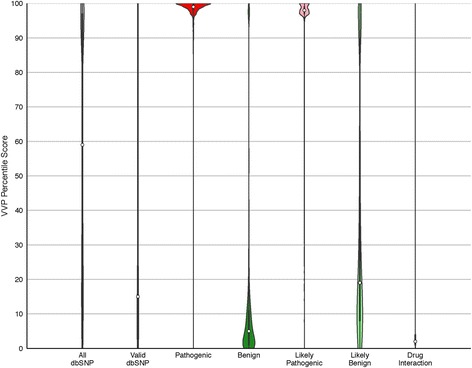
Global analysis of dbSNP using VVP. Columns are violin plots wherein the width (x-axis) of the shape represents a rotated kernel density plot. Boxplots lie within the violins with white dots denoting the median VVP score; solid black bars representing the interquartile range (IQR), and the thin black lines corresponding to 1.5 * IQR. The far left-hand (grey) column summarizes the results for the entirety of dbSNP. The remaining columns represent the data by ClinVar category. All variants were scored as heterozygotes (VVP Dominant model). All: entirety of dbSNP (155,062,628 variants, mean score: 60). valid: all variants with valid status in dbSNP (1,402,274 variants, mean score: 35). Pathogenic: all ClinVar pathogenic variants in dbSNP (33,693, mean score: 93). Benign: all ClinVar benign variants in dbSNP (21,443, mean score: 19). Likely Pathogenic: ClinVar variants annotated as likely pathogenic (7587, mean score: 92). Likely Benign: ClinVar variants annotated as likely benign (36,719, mean score: 41). Drug Interaction: dbSNP variants implicated in drug response (230, mean score: 45). Additional file 2: Figure S2 provides plots CADD and SIFT for the pathogenic and benign portions of dbSNP

Next, we used VVP to score the entire contents of dbSNP [12]. Consistent with the benchmarks presented in Table 1, this compute required only 82 s of CPU time using a 40-core server with network storage. Figure 4 summarizes the VVP scores for the ~ 155 million human variants from dbSNP Build 146, broken down by category. The results of this compute are displayed as violin plots wherein the proportion of variants with a given VVP percentile score determines the width of the plot. All variants were scored as heterozygotes; therefore, these results do not take zygosity into account. The far right-hand column of Fig. 4 summarizes the results for the entirety of dbSNP. For all of dbSNP, 53% of variants have scores > 56, whereas for the portion of dbSNP marked as validated only 27% of variants exceed a VVP score of 56.

The remaining columns in Fig. 4 distribute these results by ClinVar category. The reciprocal natures of the benign and pathogenic distributions in Fig. 4 provide a high-level overview of the ability of VVP to distinguish benign and pathogenic variants, even in the absence of zygosity information. Equally consistent trends are seen for the likely benign and likely pathogenic classes, although, as would be expected, the separation is less pronounced. Similarly, the plot for the validated portion of dbSNP variants indicates that most are neutral (median score 15, mean score 35). Finally, the drug response category is also notable for its high percentage of neutral variants (median score 21), despite their known roles in drug response. This finding is discussed in more detail below.

### Using percentile scores for VUS interpretation

VVP Percentile scores have several useful and intuitive features designed to speed interpretation of variants of unknown significance (VUS). VVP percentile scores range from 0 (least damaging) to 100 (maximally damaging), with 50 being the expected score for a neural variant, and scores greater than 57 indicating high impact on gene function with a false discovery rate of less than 0.0644 on ClinVar, and 4.0% on a WGS. See Figs. 2, 3 and Table 3 respectively.

VVP percentile scores have another important feature: they control for the fact that some genes exhibit more variation than others. For example, rare variants inducing non-conservative amino acid changes at conserved positions within the *BRCA2* gene are relatively common compared to *CFTR*, a fact documented in Fig. 1. Renormalizing the raw scores to percentile ranks adjusts for this. This means that a coding variant in *CFTR* with a percentile score of 65 can be directly compared to one in *BRCA2* with a percentile score of 80, with the *CFTR* variant predicted to be the less damaging of the two. Note that this is possible because of VVP’s flat J curve (Fig. 3), which demonstrates that the comparison can be made because accuracy of VVP for a score of 80 and a score of 65 are nearly equal, yet another illustration of the importance of considering J when interpreting prioritization scores. These sorts of within-class comparisons can also be made for non-coding and intergenic variants; for example, a synonymous variant in *CFTR* with a percentile score of 75 can be directly compared to a *BRCA2* UTR variant, as both of these variants belong to the same VVP effect class: non-coding.

Comparing the percentile ranks of variants belonging to different classes is not advisable, as percentile scores measure a variant’s severity only within that class. Raw scores should be used instead. To see why, consider an intergenic variant with a percentile rank of 95. This means its raw score is among the top 5% for all intergenic variants in gnomAD data. Thus, this variant is likely a rare change at a highly conserved intergenic site. Nevertheless, its raw score will usually be less than a stop-codon inducing coding variant with the same percentile rank, as an equally rare, conserved nonsense variant will have a greater *h*_*i*_/*a*_*i*_ ratio (See Eq. 2 and REFS [13, 14] for additional details). This fact simply reflects the preponderance of coding alleles compared to non-coding alleles with known pathogenic effects.

**Fig. 5:**
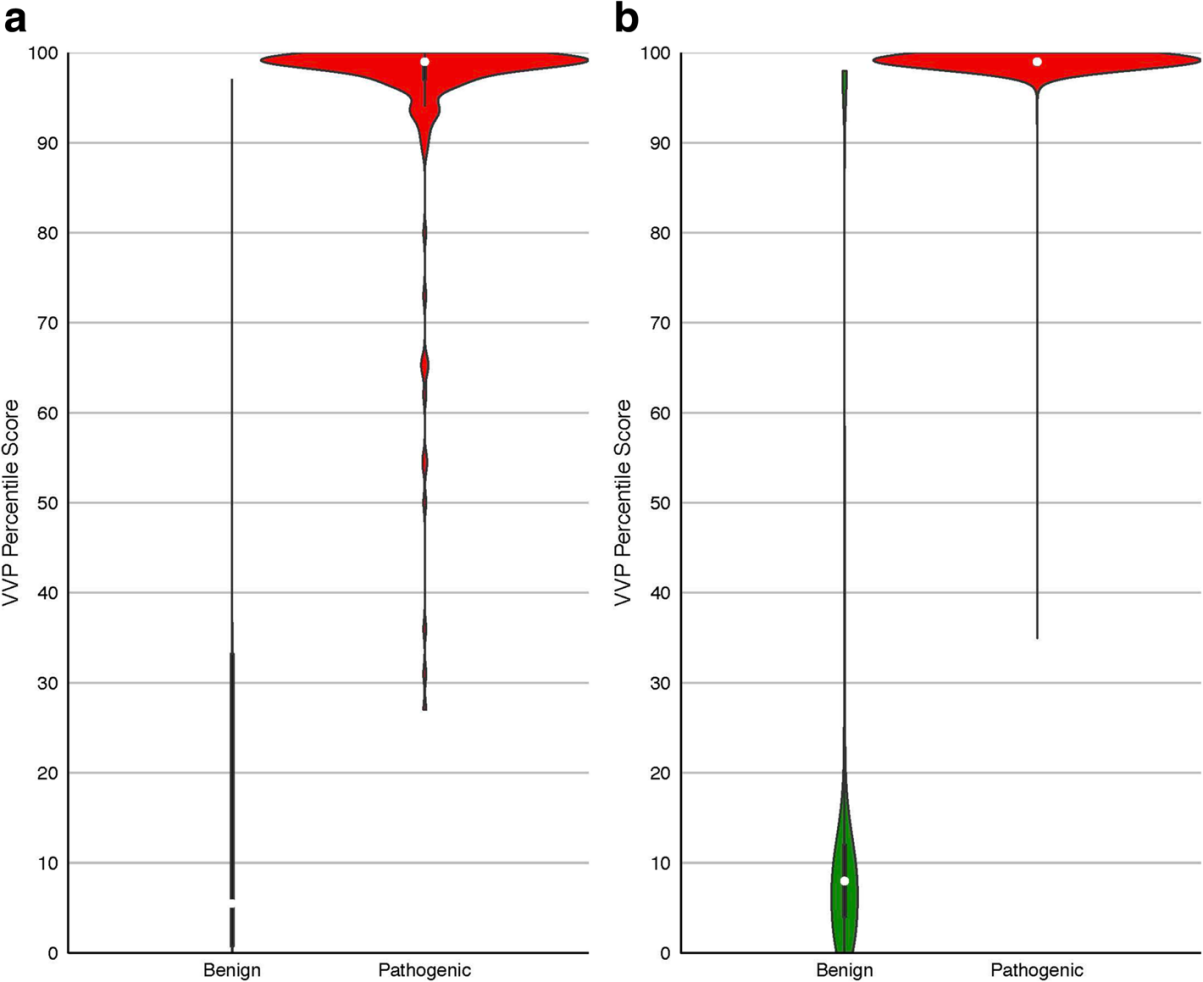
VVP percentile scores for ClinVar CFTR and BRCA2 variants. Violin and box plots are described in Fig. 4. Percentile Scores are shown on the y-axis; benign variants on the left, pathogenic on the right. **a** CFTR. Pathogenic: 897 variants, mean score: 100. Benign: 466 variants, mean score: 17. **b** BRCA2. Pathogenic: 249 variants, mean score: 93. Benign: 6 variants, mean score: 34. All scores were generated without using genotype information, i.e. the variant was scored as a heterozygote

Figure 5a presents the distribution of percentile scores for all benign and pathogenic CFTR ClinVar variants. These data are displayed as violin plots, wherein the width of each plot is proportional to the number variants with a given VVP percentile score. The left half of each panel in Fig. 5 shows the distribution for benign ClinVar variants, the right half pathogenic ones. As can be seen, CFTR pathogenic variants generally have high percentile scores.

### VVP errors

Although known pathogenic variants generally have high VVP percentile scores, (c.f. Figs. 4 and 5), VVP may fail to assign a pathogenic variant a high score when it is located in a unique functional site not accounted for by the components of VAAST’s LRT model. These cases are false negatives. VVP may also place high percentile and raw scores on some known benign variants (false positives). These cases arise when a variant is rare or absent from the background data (gnomAD), either through insufficient sampling of a site, high levels of no-calling, or because of population stratification, which can make what is a major allele in one ethnic group appear to be (erroneously) rare in the general population, leading to higher VVP scores. As more WGS data becomes available, these types of errors will decline in frequency.

VVP may also place low percentile and raw scores on some types of known pathogenic variants. These cases are also not errors, but rather reflect the catchall nature of how the term ‘pathogenic variant’ is used. Problematic examples include common disease-causing alleles with low effect sizes, pharmacogenomics (drug response) variants, and alleles under balancing selection, or at high frequency in the population due to genetic drift. These situations are discussed in more detail in the following paragraphs.

### Common disease and drug response

Common disease-causing variants and/or alleles with low relative risk will usually receive moderate percentile scores compared to high-impact Mendelian disease-causing variants. This phenomenon is well illustrated by drug response variants in Fig. 4; these variants are often common, are predicted to have low impact on gene function, and may have no impact on patient health until challenged by a particular drug that often does not exist in nature. Their lower VVP scores (median 21, mean 45) reflect these facts. Better means to identify and prioritize such variants is a difficult problem, and is an unmet need in variant prioritization.

### Balancing selection and bottleneck effects

Balancing selection (heterozygote advantage) may also act reduce the raw scores and percentile ranks of known pathogenic variants. Population bottlenecks that fortuitously increase a damaging variant’s population frequency will also depress VVP scores. The *CFTR* locus is notable in this regard. Recessive cystic fibrosis causing pathogenic alleles occur in approximately 1/25 Europeans. The high frequency of these alleles is thought to be due to balancing selection, as heterozygous individuals may have a survival advantage during typhoid fever epidemics [33].

In cases of balancing selection, the raw and percentile VVP scores will reflect this fact: variants that are beneficial as heterozygotes will generally have lower scores but will be scored as pathogenic when homozygous. F508del (the most common disease-causing CFTR allele) for example, is present in 20 of the 1000 Genomes Project phase 3 individuals. When scored by VVP as a heterozygote using gnomAD, it has a raw score of 10.98 and its percentile rank is 12 (non-damaging/protective). When homozygous, its VVP raw score is 24.65, and its VVP percentile score is 97, highly damaging. Thus, its heterozygous score reflects its protective role, and its homozygous VVP score, its recessive pathogenic nature. In fact, Fig. 5a suggests that F508del may not be the only *CFTR* allele under balancing selection: although the majority of pathogenic *CFTR* variants are well distinguished from benign alleles by VVP score, the tail of the *CFTR* pathogenic distribution is notably extended downward compared to the pathogenic *BRCA2* distribution shown in Fig. 5b. This phenomenon is a consequence of the higher allele frequencies characteristic of *CFTR* pathogenic variants. The shape of the benign distribution is a consequence of the small number benign *CFTR* variants in ClinVar, e.g. only 9. This ability of VVP to provide prioritization scores for the variant in either a homozygous or heterozygous state is designed to speed clinical interpretation of VUS.

### Zygosity and interpretation

One of the most underappreciated aspects of variant prioritization is the relationship between variant effect, zygosity, and disease. Truly recessive alleles when heterozygous have no negative impact on health no matter how damaging the variant’s impact upon gene function, whereas even mildly damaging alleles in heterozygotes may prove lethal in homozygotes. This should always be kept in mind when interpreting VVP’s variant prioritization scores. VVP explicitly models zygosity, and its default outputs contain raw and percentile scores for both heterozygous and homozygous cases*.* For example, CFTR F508del [23] when scored as a heterozygote has a minimal impact, whereas the homozygote has a highly damaging percentile score see Fig. 1.

## Conclusions

VVP is easy to use, and integrates sequence conservation, the type of sequence change, allele frequencies, zygosity and gene-specific burden, all in a single unified scoring scheme. Our demonstrations using the *BRCA2* and *CFTR* genes serve to illustrate how this approach can powerfully inform the diagnostic prioritization process, speeding and simplifying interpretation.

VVP is ultra-fast, and can easily scale to cohorts of many thousands of whole genomes and large population-scale collections of variants. To illustrate this, we used VVP to prioritize the entirety of dbSNP, some 155 million variants. That compute required 95 min on a machine with 4 cpus and 16 GB of RAM.

Our ClinVar and WGS benchmarks further demonstrate the utility of VVP’s approach to prioritization. These analyses also illustrate an important and poorly recognized aspect of variant prioritization: a tool can perform well in low-throughput diagnostic use-case scenarios, but still be poorly suited for high-throughput applications that rely upon filtering variants, because of the shape of its J curve. Because VVP’s J-curve is nearly flat for percentile scores between 20 and 90 for both coding and non-coding variants, users can move score thresholds up or down within this range, with little loss of accuracy. This is less true of SIFT and CADD (Fig. 3). This property of VVP percentile scores makes them especially useful for filter-based work flows. The constancy of VVP’s performance characteristics for both diagnostic and WGS applications together with its speed are thus major strengths for large-scale WGS analyses; and prerequisites for scoring the contents of large population collections such as dbSNP.

VVP is part of the VAAST package [13, 14], is free for academic use, and a community-moderated mailing list is available. Located at https://github.com/Yandell-Lab/VVP-pub.

VVP is for research purposes only.

## Availability and Requirements

**Project name**: VVP.

**Project home page**: https://github.com/Yandell-Lab/VVP-pub

**Operating system(s)**: Platform independent.

**Programming language**: C.

**Other requirements**: none.

**License**: Open Source Initiative-compatible MIT license.

## Additional files

Additional file 1: **Figure S1.** ROCs for ClinVar using various VVP impact scoring schemes. Top: coding variants. Bottom: non-coding. CADD is shown for reference purposes and for ease of comparison to Fig. 2. Data and Command lines are exactly as in Fig. 2, except for alterations to VVP impact scoring as denoted. (PDF 115 kb)

Additional file 2: **Figure S2.** Violin plots for the ClinVar dataset. Scores have been normalized as in Fig. 3. Note how the VVP benign and pathogenic scores are better separated. (PDF 157 kb)

Additional file 3: **Figure S3.** Mean scores broken down by allele frequency for VVP, CADD and SIFT. Data are for NA12878 WGS. Note very non-linear nature of the VVP curve compared to CADD and SIFT. As a result, VVP will rarely assign a common variant a high score. A desirable feature for high throughput WGS-driven analyses aimed at identification of rare, Mendelian alleles. (PDF 74 kb)

Additional file 4: **Figure S4.** CADD box plots for all ClinVar and NA12878 variants broken down by CADD scoring class. These results help to explain CADD’s call rate on NA12878. Note that CADD assigns high scores to FRAME_SHIFT and STOP_GAINED variants in both ClinVar and NA12878. Score > 23 is threshold for damaging. (PDF 152 kb)

Additional file 5: **Figure S5.** VVP box plots for all ClinVar and NA12878 variants broken down by CADD scoring class. Note that in contrast to CADD’s scores for these same variants (see Additional file 4: Figure S4), VVP assigns high scores to FRAME_SHIFT and STOP_GAINED variants in ClinVar, but low scores for those same classes in NA12878. ClinVar scored as in Fig. 2a. NA12878 was scored using the observed zygosity of each variant. Score > 56 is threshold for damaging. (PDF 177 kb)

## Abbreviations

ACMG: American College of Medical Genetics and Genomics
AUC: Area Under (ROC) Curve
BRCA1: Breast Cancer Associated Gene 1
BRCA2: Breast Cancer Associated Gene 2
CFTR: Cystic fibrosis transmembrane conductance regulator Gene
CRD: Cumulative Rank Distribution
INDEL: Insertion or deletion variant
LRT: Likelihood Ratio Test
NHS: National Health Service (UK)
ROC: Receiver Operator Curve
SNV: Single Nucleotide Variant
VAAST: Variant Annotation, Analysis, and Search Tool
VUS: Variants of Uncertain Significance
VVP: the VAAST Variant Prioritizer
WGS: whole Genome Sequence
