# Supplementary figures and images for: The VAAST Variant Prioritizer (VVP): ultrafast, easy to use whole genome variant prioritization tool
Source: BMC Bioinformatics. 2018 Feb 20;19:57. doi: 10.1186/s12859-018-2056-y (PMC5819680; doi:10.1186/s12859-018-2056-y)

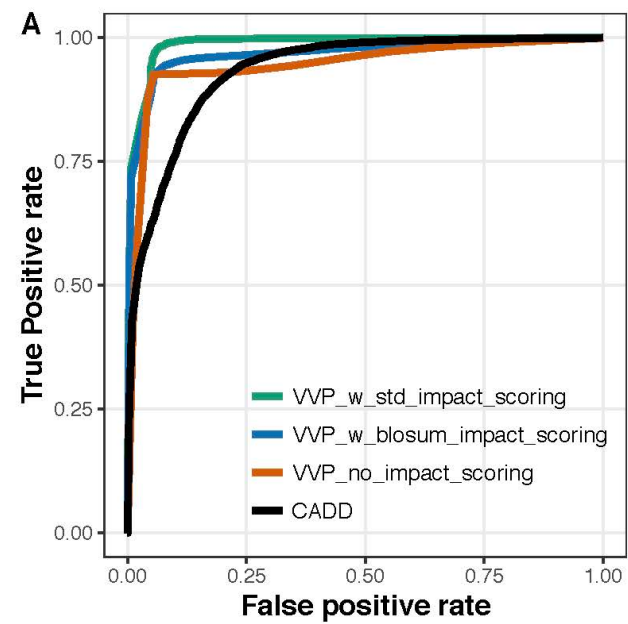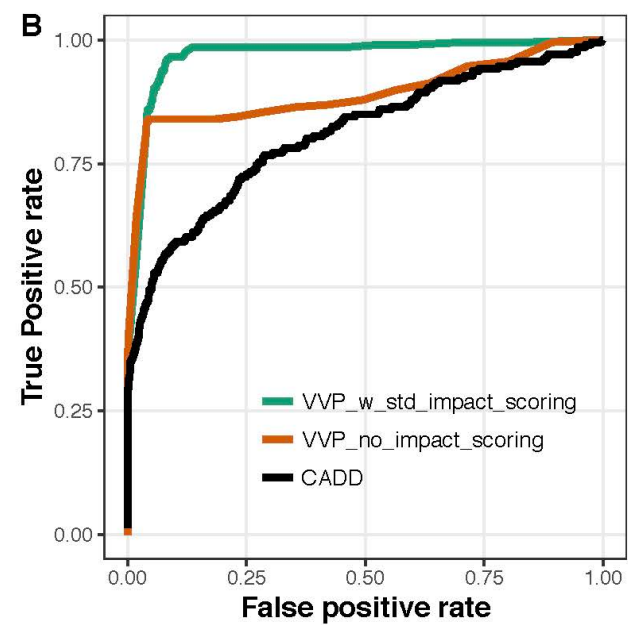

Supplement: Supplementary file 1 — Figure S1. ROCs for ClinVar using various VVP impact scoring schemes. Top: coding variants. Bottom: non-coding. CADD is shown for reference purposes and for ease of comparison to Fig. 2. Data and Command lines are exactly as in Fig. 2, except for alterations to VVP impact scoring as denoted. (PDF 115 kb) [file 12859_2018_2056_MOESM1_ESM.pdf]

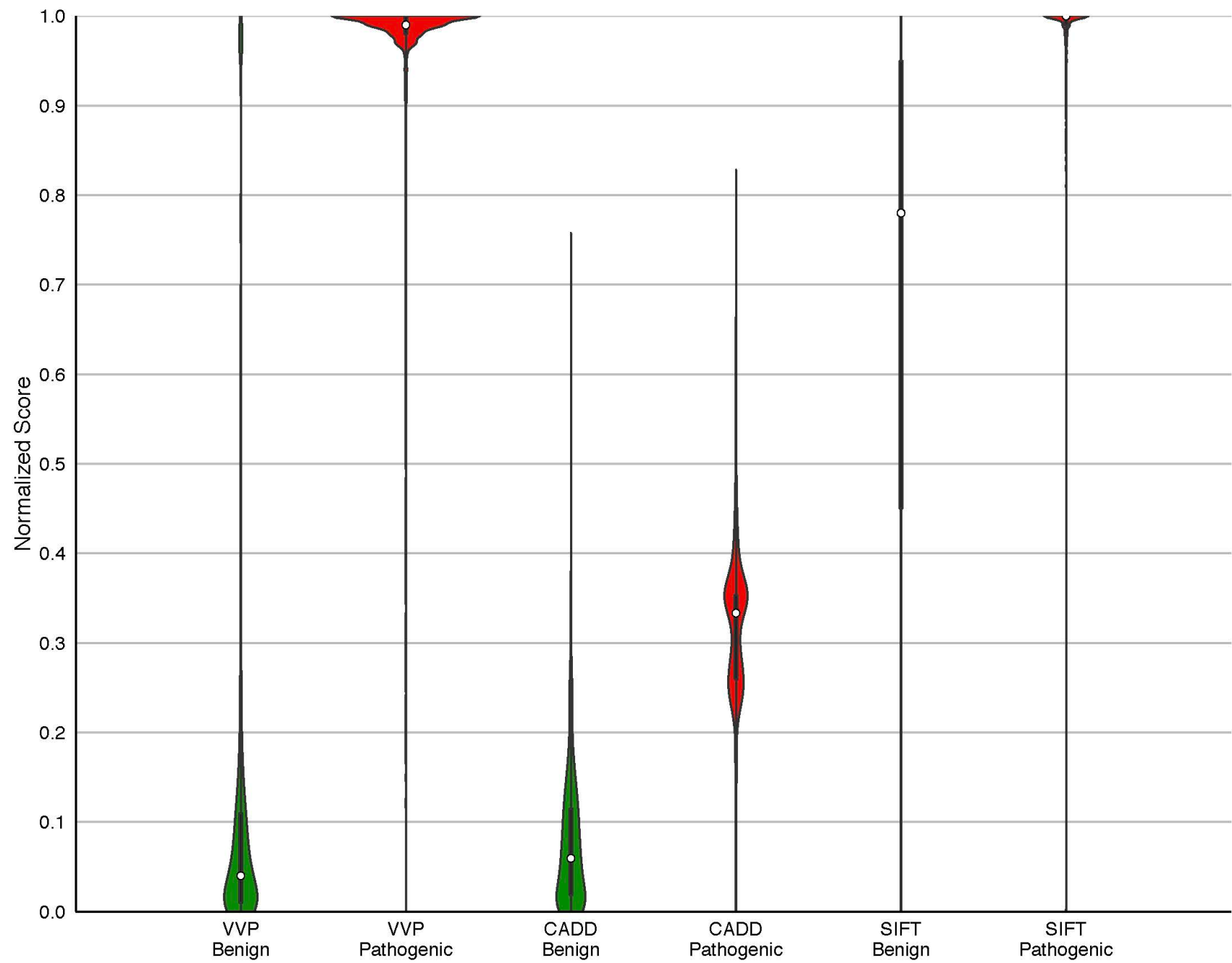

Supplement: Supplementary file 2 — Figure S2. Violin plots for the ClinVar dataset. Scores have been normalized as in Fig. 3. Note how the VVP benign and pathogenic scores are better separated. (PDF 157 kb) [file 12859_2018_2056_MOESM2_ESM.pdf]

**VVP AF v Mean Combined Score**

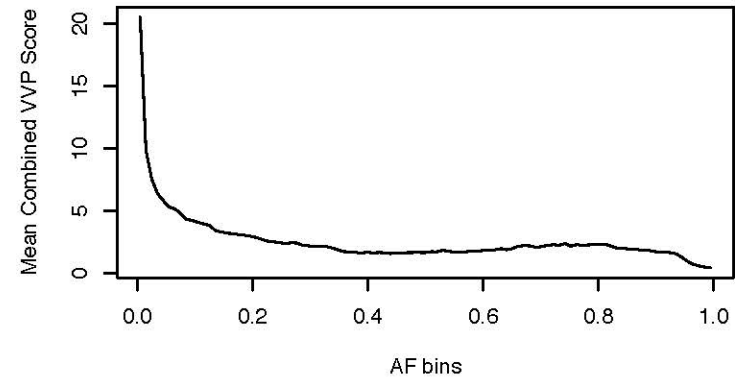

**CADD AF v Mean score**

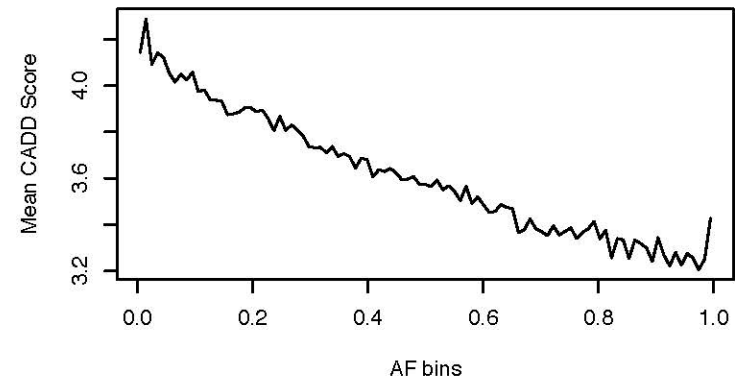

**SIFT AF v Mean score**

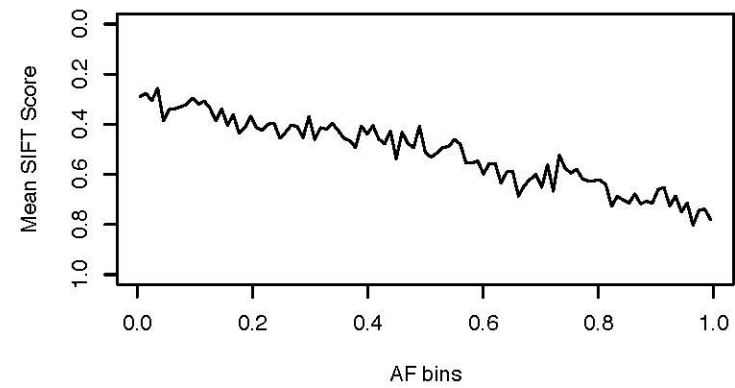

Supplement: Supplementary file 3 — Figure S3. Mean scores broken down by allele frequency for VVP, CADD and SIFT. Data are for NA12878 WGS. Note very non-linear nature of the VVP curve compared to CADD and SIFT. As a result, VVP will rarely assign a common variant a high score. A desirable feature for high throughput WGS-driven analyses aimed at identification of rare, Mendelian alleles. (PDF 74 kb) [file 12859_2018_2056_MOESM3_ESM.pdf]

### Clinvar – CADD

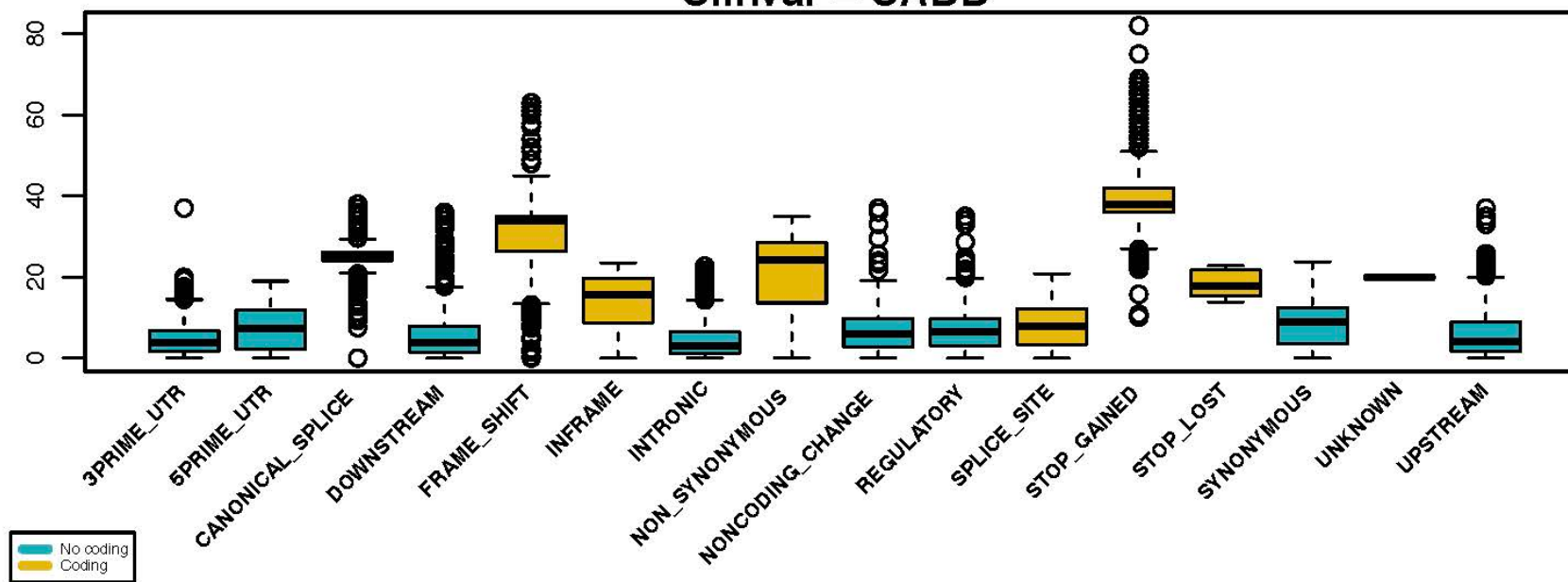

### NA12878 – CADD

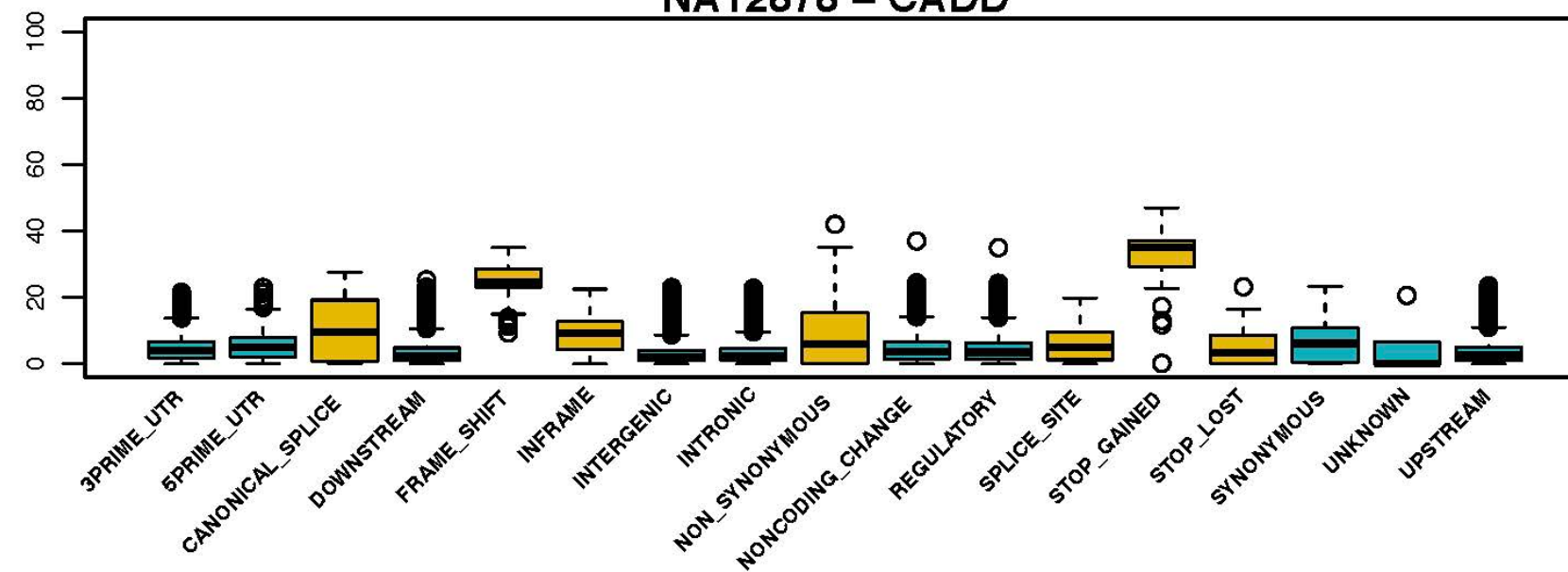

Supplement: Supplementary file 4 — Figure S4. CADD box plots for all ClinVar and NA12878 variants broken down by CADD scoring class. These results help to explain CADD’s call rate on NA12878. Note that CADD assigns high scores to FRAME_SHIFT and STOP_GAINED variants in both ClinVar and NA12878. Score > 23 is threshold for damaging. (PDF 152 kb) [file 12859_2018_2056_MOESM4_ESM.pdf]

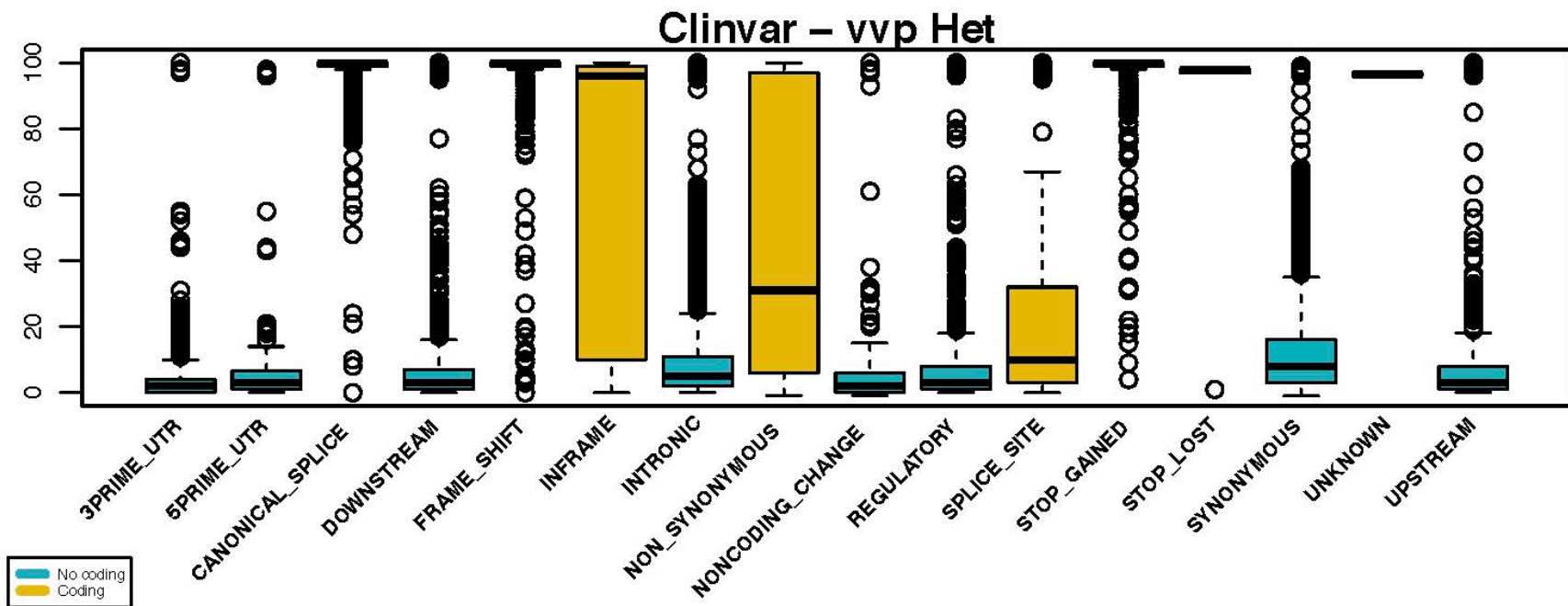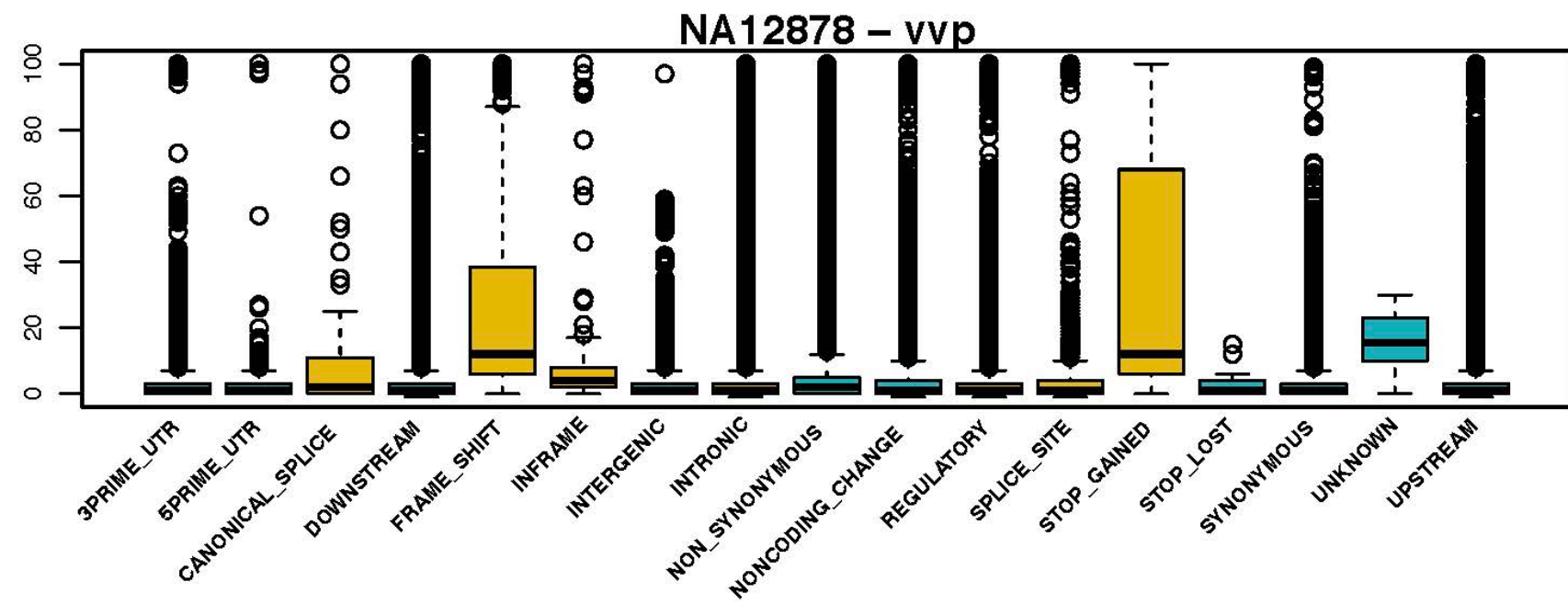

Supplement: Supplementary file 5 — Figure S5. VVP box plots for all ClinVar and NA12878 variants broken down by CADD scoring class. Note that in contrast to CADD’s scores for these same variants (see Additional file 4: Figure S4), VVP assigns high scores to FRAME_SHIFT and STOP_GAINED variants in ClinVar, but low scores for those same classes in NA12878. ClinVar scored as in Fig. 2a. NA12878 was scored using the observed zygosity of each variant. Score > 56 is threshold for damaging. (PDF 177 kb) [file 12859_2018_2056_MOESM5_ESM.pdf]
